# Yield and Technological Quality of Pirarucu Trimming Surimi According to the Number of Washing Cycles, Rice Flour Content, and Cooking Temperature

**DOI:** 10.3390/foods12142748

**Published:** 2023-07-19

**Authors:** Gabriella Leite Magalhães, Manoel Soares Soares Júnior, Márcio Caliari, Maria Lúcia Guerra Monteiro, Eliane Teixeira Mársico

**Affiliations:** 1 School of Agronomy, Federal University of Goias (UFG), Goiania 74690-900, Brazil; gabriellaleite@discente.ufg.br (G.L.M.); manoel@ufg.br (M.S.S.J.); macaliari@ufg.br (M.C.); 2 Institute of Chemistry, Federal University of Rio de Janeiro (UFRJ), Rio de Janeiro 21941-909, Brazil; 3 Department of Food Technology, Fluminense Federal University (UFF), Rio de Janeiro 24220-000, Brazil; elimarsico@gmail.com

**Keywords:** *Arapaima gigas*, *Oryza sativa* L., by-product use, fish paste, nutritional and functional characteristics

## Abstract

This research aimed to optimize surimi production with innovative raw materials called pirarucu trimmings and broken rice grains, which are by-products from food industries. The effects of three independent variables (number of washing cycles, content, and cooking temperature of rice flour—RF) on surimi’s physical, chemical, and technological qualities were investigated through a Box–Behnken design. The number of washing cycles affected yield (77–93%), moisture (55–67%), lipids (18–35%), protein (7.15–11.88%), whiteness (46.73–64.45), chroma (8.86–13.18), hue angle (80.79–93.12°), cohesiveness (0.40–0.61), springiness (0.51–0.99), and freeze stability after 4 weeks (85.16–96.53%). RF concentration affected moisture, lipids, cohesiveness, springiness, and freeze–thaw stability after 4 weeks. RF cooking temperature affected moisture, chroma, cohesiveness, and springiness. The optimal conditions for surimi production with high yield and overall quality were three washing cycles, 6% of RF, and RF cooking temperature at 85 °C. It reveals the promising potential of both by-products to be used as an ingredient in restructured products and contribute to improving agri-industry sustainability.

## 1. Introduction

Brazil has performed excellently in fish production, such as the promising Amazonian pirarucu (*Arapaima gigas*). In 2021, the country produced 1859 tons of pirarucu in captivity (preliminary data). The largest producer in the north region is Rondônia, with almost 1000 tons, followed by Tocantins with 227 tons, and Pará with 221 tons. This production has supplied the market as there is a fishery decline [1].

Pirarucu is usually consumed as fillets or dry-salted, and its significant and promising consumption in both domestic and foreign markets is related to its attractive pinkish color, mild and pleasant flavor, and boneless meat, thus being one of the best-evaluated native fishes for consumption [2]. However, during the production of pirarucu fillets, 30 to 50% of the fish is used; the remainder gives rise to several by-products, such as carcass, offal, skin, and trimmings [3]. Moreover, preparing pirarucu trimming surimi could be a way of using by-products from manufacturing.

Although freshwater fish myofibrillar proteins have low gelation properties [4], some studies have developed surimi using these species, such as silver carp [5,6,7] and Chinese carp [8]. Nevertheless, there are no records of the use of pirarucu or its by-products to obtain surimi.

The number of washing cycles and starch addition are some factors that govern the quality and physical properties of surimi. Washing contributes to the technological and sensorial quality of the final product, being indispensable during preparation due to the removal of sarcoplasmic proteins, myofibrillar protein concentration, and, consequently, fat removal [9].

Starch contributes to gel springiness due to protein–polysaccharide interaction, neutralizing the reduction in springiness caused by decreased myofibrillar protein in meat emulsions. Also, starches act through filling empty spaces, covering the surface of the protein network, or being trapped inside the protein network [10]. Starch granules directly interfere with the gel matrix, for when heated, the granules become larger due to water absorption, leading to the growth of a surimi–starch system [11].

Wheat and potatoes constitute the most widely used starch sources to make surimi [6,12], as well as sweet potatoes [11] and cassava [9,13]. During rice processing, by-products such as broken grains are commonly used for animal feed. However, as raw materials, they can be used to manufacture rice flour (RF), a promising starch source [14].

This study aimed to determine the yield and evaluate the physical, chemical, and technological quality of pirarucu trimming surimi according to the number of washing cycles, RF content, and cooking temperature to enhance industrial by-products and provide information on a novel ingredient suitable for processing restructured products.

## 2. Materials and Methods

### 2.1. Obtaining Raw Material, Ingredients, and Reagents

**Figure 1:**
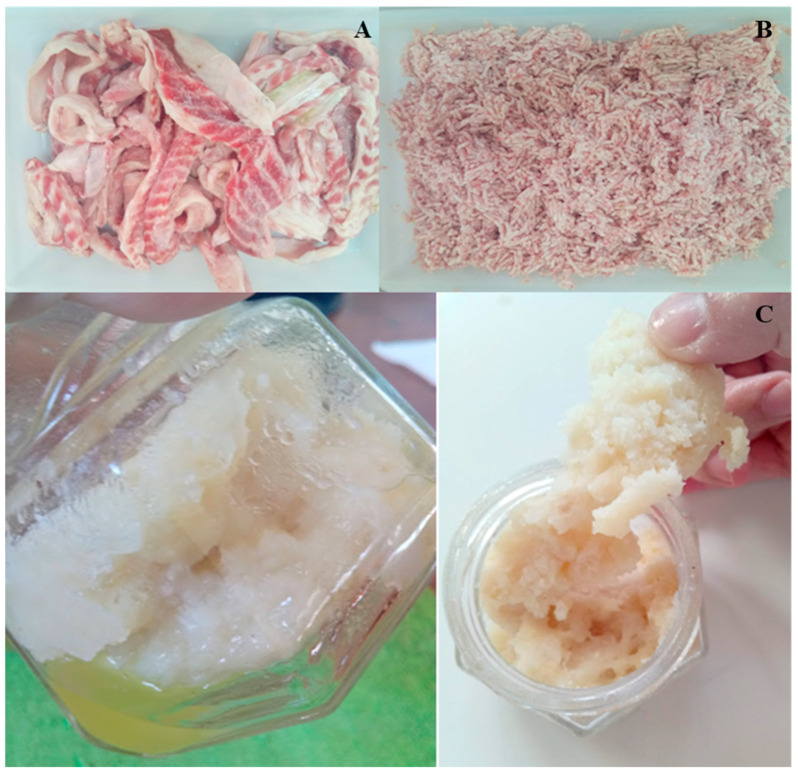
(**A**) Pirarucu (*Arapaima gigas*) trimmings; (**B**) pirarucu meat samples after disintegration; (**C**) pirarucu (*Arapaima gigas*) surimi with three washing cycles, 8% rice flour, and heating at 95 °C for 30 min to gelatinization.

The pirarucu trimmings (Figure 1A) were donated by Peixe Bom Pescados, located in Goianésia, Goiás, Brazil. They were immediately transported to the processing plant in an isothermal box with artificial ice at 5 ± 1 °C, ground with a 5 mm disc in an electric meat grinder (Omega, TA 32x, Cardano Al Campo, Italy) (Figure 1B), packed in polyethylene bags, and stored in a freezer at −18 °C until being analyzed and processed.

The broken rice grains were kindly provided by Cristal Alimentos, a rice processing industry located in Aparecida de Goiânia, Goiás, Brazil. They were ground in a cyclone rotor mill (Tecnal, TE-651/2, Piracicaba, Brazil). After grinding, the flour was sieved in an 80-mesh sieve, packed in polyethylene bags, and stored in a freezer at −18 °C, until analysis and use in experimental products. The cryoprotectants used were crystal sugar (Cristal Alimentos^®^), refined salt, stabilizer, and emulsifier for cooked meat products consisting of sodium tripolyphosphate (INS 451i) and potassium polyphosphate (INS 452ii) (Kerry^®^, Três Corações, Minas Gerais). All reagents used in the physicochemical analyses were from Synth^®^ and presented purity analysis (P.A.).

### 2.2. Proximate Composition of Raw Materials and Surimi

Determination of moisture via oven at 105 °C (925.09–1925), protein via micro-Kjeldahl (920.87–1920), lipid via Soxhlet with the use of petroleum ether solvent (920.39–1920), ash in a muffle oven at 550 °C (923.03–1923), and carbohydrate according to difference in content of pirarucu trimmings and RF, as well as moisture, protein, and lipid levels in pirarucu trimming surimi was carried out according to the methods described in AOAC [15] in triplicate.

### 2.3. Thermal and Pasting Properties of RF

Thermal properties were set using a differential scanning calorimeter (DSC, TA Instruments, Q20, Newcastle, UK) according to the methodology described by Jia et al. [12]. The sample was weighed (around 2.2 mg dryly basis) in an aluminum sample holder, and then 6 µL of distilled water was added. The sample holder was sealed in a specific press and maintained for 24 h at 25 °C to standardize water distribution. The sample was heated from 20 °C to 120 °C at a rate of 10 °C/min, and an empty sample holder was used as a reference. Gelatinization temperatures (onset, peak, and conclusion) and enthalpy variation (ΔH) during heating were determined using TA Universal Analysis software (TA Instruments, UK).

Pasting properties were determined using Rapid Visco Analyzer (Perten, RVA 450, Huddinge, Sweden) according to the 162 “Rapid Pasting Method using the Newport Rapid Visco Analyzer” methodology of the International Association for Cereal Science and Technology [16]. A suspension of 3.5 g sample was used in 25 mL of distilled water. The process happened at a minimum temperature of 50 °C and a maximum of 95 °C at a rate of 10 °C/min. The parameters were: pasting temperature (PT), peak viscosity (PV), final viscosity (FV), breakdown viscosity (BD), and setback viscosity (SB). Data were expressed in Rapid Visco Units (RVU) obtained through TCW3 software, Thermo Cline for Windows v3.

### 2.4. Preliminary Tests

Preliminary tests for preparing pirarucu trimming surimi were carried out to verify the starch–protein interaction during heating to promote gelation (surimi gel) [6,9]. Ground pirarucu trimmings went through 3 washing cycles using a 1:3 ratio (trimmings:water) for 20 min, 10 min stirring, and 10 min at rest, followed by the addition of 8% rice flour and homogenization using a domestic mixer (Gastromaq, BP-05, Caxias do Sul, Brazil). The water temperature used for washing was 4 °C. Then, RF and cryoprotectants (5% sucrose and 0.3% sodium polyphosphate) were added to washed pirarucu trimmings, and it was heated at 95 °C for 30 min to induce gelation. Under these conditions, it was observed that the consistency would not be adequate to obtain the final product (restructured product), as shown in Figure 1C.

In order to decrease fat separation in a second test, homogenization of the washed pirarucu trimmings, RF, and cryoprotectants (5% sucrose and 0.3% sodium polyphosphate) was performed using a cutter (Robot Coupe, R 602 V.V., Burgundy, France) for 2 min at 1400 rpm. In this test, better homogenization was verified; however, obtaining adequate consistency for applying in the restructured products was not yet possible. Because of the results obtained, and to reach appropriate characteristics, previous starch gelatinization, with a concentration of 35% (*w*/*v*), was performed, followed by its addition to the washed pirarucu trimmings. In this study, it was observed that inducing the gelation of pirarucu trimming surimi resulted in a very resistant gel, making it unfeasible to develop the restructured product. Therefore, a surimi paste was developed.

### 2.5. Box–Behnken Design to Optimize Surimi Quality

A Box–Behnken experimental design [17] was used to minimize the experiments, evaluating the effect of the chosen variables and the experimental error. The independent variables were the number of washing cycles (X_1_), RF concentration (X_2_), and RF cooking temperature (X_3_), while the dependent variables were the physicochemical and technological properties of pirarucu trimming surimi. Each independent variable was studied at three levels (+1.0 and −1), totaling 15 experiments (Table 1).

The levels were chosen according to previously carried out studies on washing cycles [9], RF concentration [6], and RF cooking temperature [18]. The pirarucu trimmings were thawed at 4 °C for 24 h. After thawing, they were subjected to washing in a 1:3 ratio (trimmings:water). Cold running water (approximately 4 °C) was used for the washing. Then, 0.2% sodium chloride was added to each washing cycle, varying according to the experimental design. The samples were stirred in water for 10 min and then were at rest for 10 min for fat separation, being supernatant. Fat excess was removed with a slotted spoon, and water excess was removed with a fabric commonly known as “round-the-world fabric” used for cheese pressing.

Yield related to the washing cycle was performed through the initial weight of pirarucu trimmings and the weight after washing, using Equation (1).

(1) $$\mathrm{Yield}\;(\%)=\frac{\mathrm{Final}\;\mathrm{weight}}{\mathrm{Initial}\;\mathrm{weight}}\;\times \;100$$

RF cooking, using 35% (*w*/*v*), was induced according to the design employing a digital water bath (Tecnal, TE-056-MAG, Piracicaba, Brazil) and heated for 15 min. After heating, the samples were quenched for 5 min to stop cooking. The cooked RF was added to the already-washed pirarucu trimmings following the addition of cryoprotectants (5% sucrose and 0.3% sodium polyphosphate) and homogenization with a cutter for 2 min at 1400 rpm (Robot Coupe, R 602 VV., Burgundy, France). Subsequently, the samples were packed in plastic bags and frozen at −18 °C, thus obtaining frozen “surimi”. Responses like yield, moisture, proteins, lipids, colorimetric analysis, texture profile, and freeze–thaw stability were obtained for each test.

### 2.6. Instrumental Color Analysis of Surimi

The color of the samples was measured with a colorimeter (Konica Minolta, BC-10, Osaka, Japan) using the CIELab scale (L*, a*, and b*). Color measurement was expressed in terms of luminosity L* [0 (black) to 100 (white)] and chromaticity parameters a* [green (−) to red (+)] and b* [blue (−) to yellow (+)]. Based on the results of L*, a*, and b*, chroma parameters (C*) or color intensity, hue angle (H°) or hue (0° red, 90° yellow, 180° green, and 270° blue), and whiteness were calculated through Equations (2)–(4), respectively [19].

(2) C∗=a2+b212

(3) $$\mathrm{Hue}\;\mathrm{angle}\left({}^{\circ}\right){=\tan}^{-1}\left(\frac{b*}{a*}\right)$$

(4) Whiteness=L* − 3b*

### 2.7. Texture Profile

The sample was compacted into a 50 mm diameter and 25 mm height cylinder. The texture profile (hardness, gumminess, adhesiveness, springiness, cohesiveness) of surimi was determined using a TA-HD Plus Texture Analyzer (Stable Micro Systems, Godalming, UK), equipped with a 20 mm diameter cylindrical probe, Exponent Lite software, and pre-test, test, and post-test speed adjusted to 2, 1, and 5 mm/s. The trigger type was defined as automatic with a trigger force of 50 mN, a data acquisition rate of 200 pps [20], and five readings per experiment.

### 2.8. Freeze–Thaw Stability

The surimi paste was compacted up to 25 mm using a 50 mm diameter cylinder and then stored in plastic bags. The samples were frozen in a conventional freezer at −18 °C. In 1 (168 h) and 4 (672 h) weeks, they were thawed at 4 °C overnight. Drip calculation was performed according to the drip weight rate after frozen storage concerning the total sample weight [12] (Equation (5)).

(5) $$\mathrm{Thawing}\;\mathrm{drip}\;\left(\%\right)=\frac{\mathrm{Drip}\;\mathrm{weight}\;(g)}{\mathrm{Sample}\;\mathrm{weight}\;(g)}\;\times \;100$$

### 2.9. Statistical Analysis

Results were analyzed using variance and multiple regression and visualized via response surface graphs. The adequacy of the model equations was expressed using the coefficient of determination (R^2^), and the F test determined statistical significance. The experimental design was developed and analyzed using Statistica^®^ 7.0 software (Statsoft^®^, version 7.0, Tulsa, OK, USA).

## 3. Results

### 3.1. Proximate Composition, RF Thermal, and Paste Properties

The flour presented the following contents: moisture—9.06 ± 0.11%, lipids—1.28 ± 0.04%, protein—9.26 ± 0.15%, ash—0.96 ± 0.04%, and carbohydrates—88.49 ± 0.22%.

In Brazilian legislation, there are no identity and quality standards related to the proximate composition for RF, only moisture for wheat flour, which must be a maximum of 15% [21], thus indicating compliance with the RF. Besides being an essential factor for flour storage, with this moisture content, it is possible to store it at room temperature with no damage to the product [22]. Moisture also influences flour viscosity, as the higher the moisture, the lower the viscosity [23].

Pereira et al. [24] reported a lipid content of 1.57% for RF, similar to that found in this study. According to Lin et al. [25], rice contains around 1–3% lipids. Lipid content also influences viscosity through simultaneous interactions. The amylose–lipid complex formation is responsible for the viscosity increase. However, due to its lubricating effect, lipids (or the increase in lipid content) can decrease viscosity [23].

Protein molecules directly interfere with the properties of the flour paste, for together with starch, they form a binding domain responsible for greater water retention capacity [26]. According to Lin et al. [25], rice contains around 8% proteins. Fitriani et al. [22] found a content of 8.48%, whereas Ascheri et al. [27] found 9.89% of protein. Such variation can be explained since protein content is affected by genotypic characteristics, nitrogen fertilization, solar radiation, and temperature [14].

Pereira et al. [24] and Tumuluru et al. [28] determined ash content of 1.02% and 0.93%, respectively, values close to those obtained in the present study. Tumuluru et al. [28] found a carbohydrate content of 78.69% for RF, whereas Fitriani et al. [22] obtained 80.95%. Both carbohydrate levels were lower than the values found in the present study. Carbohydrates are the main components of rice (about 90%) and correspond to starch, free sugars, and fibers [14,29].

Starches and cereal flours are typically added to meat products to improve product texture [24]. The particle size during RF cooking may be a physical barrier to heat transfer, as the greater the flour particles, the greater the physical barrier [30].

Gelatinization enthalpy is determined according to the phase transition of starch granules during heating in excess water, which changes from a crystalline to an amorphous state. The amylopectin portion is responsible for forming the crystalline region; thus, the greater the amount of amylopectin, the greater the energy used to disorder [31]. Enthalpy is also related to flour milling since the smaller the particle size, the lower the enthalpy and gelatinization temperature [32]. When comparing the particle size in this study (80 mesh) with the broken rice flour used by Setyawati et al. [31] (with greater particles of 25 mesh), this theory is confirmed, for they reached higher enthalpy (8.327 J/g) and onset (61.42 °C), peak (70.17 °C), and conclusion gelatinization temperatures (75.74 °C) than those in this study (Table 2). Furthermore, the low values found in the thermal analysis may be related to the fact that RF has many other components, such as proteins, ash, fibers, and lipids, which also tend to reduce gelatinization enthalpy [33].

Kim and Shin [34] reported RF with 80 mesh particle size, pasting temperature of 70.40 °C, peak viscosity of 308.8 RVU, breakdown viscosity of 140.7 RVU, and final viscosity of 284.5 RVU. According to these authors, the values varied as the particle size decreased. The correlation between flour pasting temperature and particle size is probably due to the greater physical barrier of larger flour particles to heat transfer and water diffusion, whereas between the final viscosity and flour particle size is probably due to the effects of other non-starchy components in grains/flour (mainly proteins and non-starchy polysaccharides) on paste viscosity and granule puffiness [32]. In comparison to the results found by Kim and Shin [34] using flour with the same granulometry (80 mesh), we observed that they obtained higher pasting temperature and peak viscosity but lower breakdown viscosity, final viscosity, and tendency to retrogradation, thus obtaining a less viscous gel than in the present study (Table 2). Higher final viscosity indicates a rigid gel structure during cooling [33]. RF contains lipids and proteins, so it tends to have a lower peak viscosity value since these components decrease expansion.

In the cooling process, amylose retrogradation begins, and with the decrease in temperature and hydrogen bonds between the molecule chains, they form again, leading to a significant increase in viscosity [25]. Kim and Shin [34] reached a tendency to retrograde 116.4 RVU, so the value found in the present study (148.13 RVU; Table 2) forms a more rigid gel during cooling since the higher the setback, the more rigid the gel formed.

Thermal and pasting properties influence surimi gelation, for when starch is added to surimi, there is a complement due to the swelling capacity of starch, thus forming a more compact and firmer surimi gel [35]. If starch has a high gelling temperature, there will be no swelling and the surimi gel strength will be lower [36], which is not the case with the RF used in this work.

Jia et al. [11] used two different types of starch, one being native sweet potato (SPS) and the other also native sweet potato, but with low pasting temperature and slow tendency to retrogradation (SR), both with different gelling temperatures, 76 and 53.4 °C, respectively. These authors observed that surimi added with SR obtained better gelling properties, as there was a protective effect on the gel structure and decreased damage to starch granules after freezing and thawing. Thus, comparing the results obtained in this research with those of the study mentioned above, RF has an intermediate gelling temperature, maintaining the gelling properties of surimi.

### 3.2. Proximate Composition of Pirarucu Trimmings

Moisture content (54.69 ± 2.33%) was higher in pirarucu trimmings, followed by lipid content (31.89 ± 0.48%), protein content (19.78 ± 0.73%), and ash content (0.71 ± 0.03%). Studies regarding the proximate composition of fish vary significantly, which impaired the comparison of our results with those found in the literature. Such variations in the proximate composition of fish can be attributed to factors such as different types of food and muscle activity, season, environment, age, and sex. Moreover, the proximate composition of pirarucu varies according to its region, which is divided into four parts: dorsal, ventral, *ventrecha* (belly), and tail [37,38,39]. Considering these works, pirarucu can have a moisture content ranging from 52.2 to 78.2%, protein content from 17.8 to 25.8%, lipid content from 1.0 to 17.1%, and ash content from 0, 9 to 1.2%, according to its different regions. In the study conducted by Martins et al. [38], the lowest moisture content found was in the *ventrecha* (belly) region, which was similar to that found in this study (54.69%). According to the authors, the *ventrecha* is the concentrated-fat region of the fish, presenting lower moisture content and, consequently, higher lipid content, which indicates that the trimmings in the present study are probably from the *ventrecha* region.

Notably, despite variations in the proximate composition, *Arapaima gigas* was considered a medium-fat fish (4–8% of body fat) in all the studies cited. Moreover, it was possible to observe that its protein content was similar to the values obtained in the present study, different from the lipid and moisture contents. Lipids and moisture are the main components that govern overall surimi quality and its preparation; accordingly, it would be necessary to adjust moisture content before adding starch, mainly for obtaining surimi gel [35]. 

### 3.3. Yield and Physical, Chemical, and Technological Quality Evaluation of Pirarucu Trimming Surimi

Experimental data for yield, moisture, proteins, lipids, color, texture, and freeze–thaw stability at 1 and 4 weeks according to the number of washing cycles, RF content, and cooking temperature are presented in Table 3 and Table 4. The adequacy of the multiple regression models was significant (*p* < 0.05) for yield, moisture, proteins, lipids, cohesiveness, springiness, hue angle, and whiteness. Moreover, in these models, the lack of adequacy was not significant (*p* < 0.05) (Table 5).

**Figure 2:**
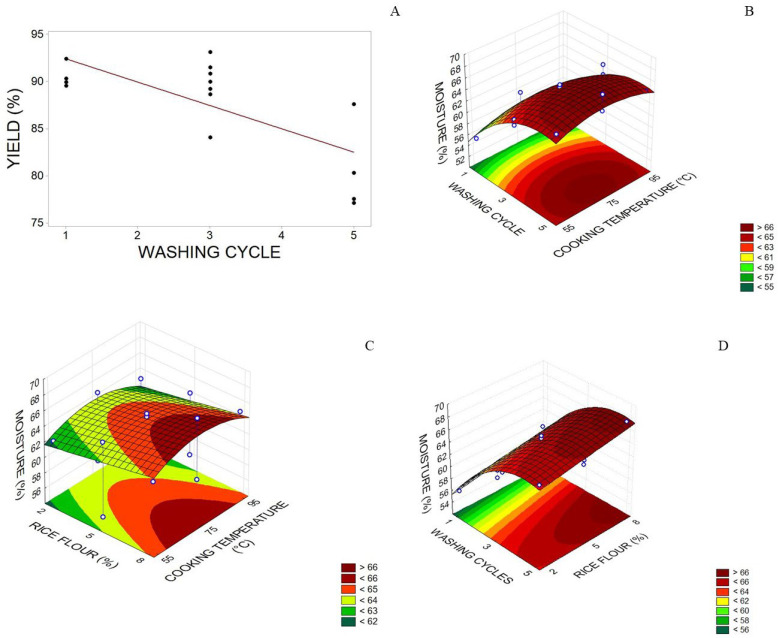
(**A**) Yield (%) of the pirarucu (*Arapaima gigas*) trimming surimi concerning number of washing cycles; (**B**) moisture (%; wet basis) to washing cycles and cooking temperature; (**C**) moisture (%; wet basis) to rice flour (RF) concentration and cooking temperature (°C) of RF); and (**D**) moisture (%; wet basis) to number of washing cycles and RF concentration. The third variable in each graphic was fixed in respective zero points.

The yield only varied (76–93%) according to the number of washing cycles (Table 5), decreasing as the number of cycles increased (Figure 2A). As expected, the washing cycle had a significant effect since yield calculation weighing was carried out before and after the washing cycle. Hamzah et al. [40] used cobia (*Rachycentron canadum*) to obtain surimi with two to five washing cycles and the same ratio of fish:washing water (1:3) in this study. They reported a yield of 61–73%. Endoma et al. [41] made surimi with *Brama orcini* obtained from three washing cycles and found a yield of 61.70 ± 0.10%, while 20.3% was achieved in surimi *Pangasius hypophthalmus* after three washing cycles [42]. That is, developing surimi with trimming of *Arapaima gigas* has a better final yield if compared to the studies cited above. The yield in surimi preparation varies according to the species of fish used, sarcoplasmic protein concentration, and the number of washing cycles [43], yet for the pirarucu trimmings used, the high lipid content could not be reduced only with washing cycles.

The model adjusted to moisture content showed a significant positive linear effect for the variables washing cycle and RF concentration and significant negative quadratic effects for the variables washing cycle and temperature. Thus, when the gelling temperature was set at 70 °C, the maximum moisture value (66%) in the graph area was found for 4 to 8% flour addition and three to five washing cycles (Figure 2B).

The response surface graphs showed that through setting the RF concentration at 5%, maximum moisture (66%) was found with an RF cooking temperature from 55 to 95 °C and three to five washing cycles (Figure 2D), whereas with the number of washing cycles set at three cycles, the maximum moisture region (66.02%) was determined with an RF cooking temperature between 65 and 85 °C and an RF concentration of 5.5 and 8% (Figure 2C).

According to Oliveira et al. [13], surimi moisture content can be influenced by washing cycles, the presence of salts in the washing solution, and also by the lack of standardization of draining operation, corroborating with this study as far as the number of washing cycles is concerned. Fogaça et al. [44] obtained for surimi with no starch variation and with 10 and 20% starch an adjusted model where moisture content increased with washing cycles and decreased with starch addition, reaching moisture levels of 65 to 80%, which was partially observed in this study, for herein moisture content increased with washing cycles and also with the addition of starch up to 8%, decreasing in higher levels when the number of washing cycles was set at three (Figure 2C), or when RF concentration was higher than 4%, the number of washing cycles was higher than three, and RF cooking temperature was set at 76 °C (Figure 2B). Moreover, only the maximum moisture value was within the range determined by these authors.

Jia et al. [12] used an Alaska pollock surimi whose moisture content was 74.5%. Liu et al. [45] obtained common carp surimi, with and without cryoprotectants, whose moisture content was 74.45 and 77.19%, respectively. Thus, adding more water would be necessary as pirarucu trimming surimi’s moisture content was lower than in the previous works. This possibly impaired starch–protein interaction and resulted in the phenomenon observed in preliminary tests, wherein gel stiffness makes it difficult to shape to prepare a restructured product. Hence, to increase the moisture content and allow for the improvement of this interaction, it is possible to increase the water ratio used during washing; this way, there is enough water available for gelling, and there is no need for starch gelatinization, which would allow the development of a surimi gel.

**Figure 3:**
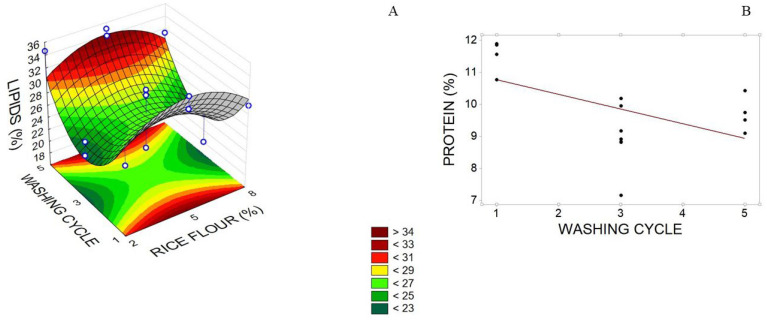
(**A**) Lipid and (**B**) protein levels in pirarucu (*Arapaima gigas*) trimming surimi concerning the number of washing cycles and rice flour (RF) concentration (%) and number of washing cycles, respectively.

The model adjusted to the lipid content of pirarucu trimming surimi showed a positive quadratic effect for the washing cycle and a negative one for RF, while RF cooking temperature did not significantly affect this response (Table 5). Minimum lipid content (22%) was found between two and four washing cycles, and RF concentration was close to 2% or 8% (Figure 3A). Due to the negative effects during surimi gelation, high lipid content in surimi processing is undesirable. According to Priyadarshana and Walpita [46], pre-washing can reduce fat content, which was observed with two washing cycles in *Amblygaster sirm*, a pelagic fish. Unsaturated fatty acids prevent non-covalent interactions of myosin, which are essential for gel formation [8].

In the model adjusted to protein, only the negative linear effect of the number of washing cycles was significant (Table 5). Regarding protein content, the values determined for pirarucu trimming surimi, with an RF concentration of 5% and three washing cycles, varied between 9 and 12% (Figure 3B), with the highest values obtained after one washing cycle and the smallest ones obtained after five washing cycles. This showed that it was possible to remove sarcoplasmic proteins since the fish trimmings had 19.78% protein.

Oliveira et al. [13] evaluated tilapia surimi (*Oreochromis niloticus*) with three washing cycles and varying cryoprotectant addition and determined a range of protein between 11.38% and 13.56%. Liu et al. [47] reported, for silver carp surimi (*Hypophthalmichthys molitrix*) with and without starch, protein contents of 18.83% and 18.06%, respectively. Both studies showed results higher than those found in this work, which can be attributed to the origin of the fish: the tilapia was farmed, while the silver carp was wild. Nevertheless, the study on farmed fish obtained values closer to those found in this study (9 to 12%).

**Figure 4:**
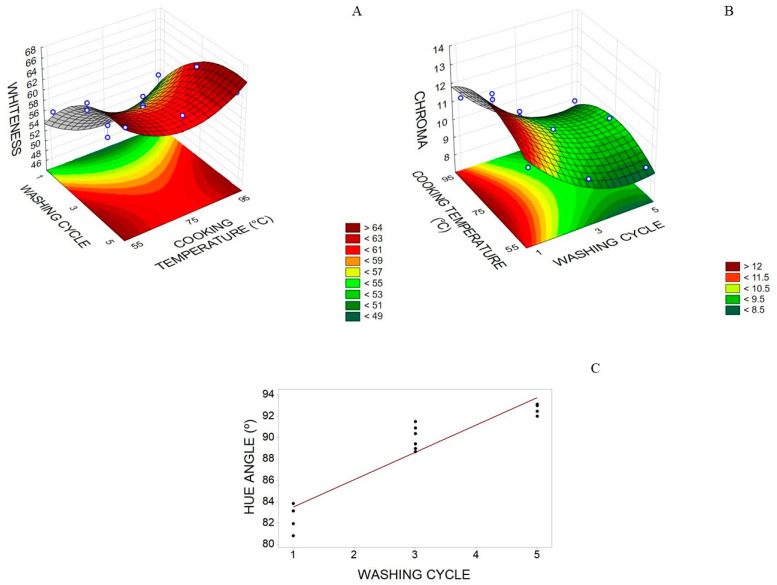
(**A**) Whiteness (%) and (**B**) chromaticity (dimensionless) of the pirarucu (*Arapaima gigas*) trimming surimi concerning number of washing cycles and cooking temperature (°C) of RF; and (**C**) hue angle (°) concerning number of washing cycles.

As far as color is concerned, there was only adequacy of the whiteness, chroma, and hue angle models. The number of washing cycles showed positive linear and negative quadratic effects, and RF cooking temperature had a positive quadratic effect on whiteness. The same significant effects for whiteness were also for chroma, yet with opposite signs (Table 5), possibly because the higher the whiteness of the sample, the less intense its color was. Only the positive linear effect of the number of washing cycles was significant for the hue angle. The area of the graph with the maximum whiteness value (64%) was between three and five washing cycles and an RF cooking temperature below 65 °C or above 85 °C (Figure 4A).

The proportional increase in sample whiteness with the increase in the number of washing cycles can be justified by the removal of sarcoplasmic proteins (myoglobin and hemoglobin) in the washing process [40]. Overall, a high whiteness value in surimi is expected, which was possible to verify with three and five washing cycles. Liu et al. [47] observed whiteness variation for silver carp surimi between 46.89 to 62, whereas in this research, whiteness values were slightly higher and varied between 50 and 64%, probably due to the fish’s natural color. According to the response surface graph (Figure 4B), the minimum chroma value (9) was obtained with three to five washing cycles and with an RF cooking temperature from 55 to 65 °C or 85 to 95 °C, correlating inversely with whiteness.

The hue angle of pirarucu trimming surimi showed values between 83 and 93 °C (Figure 4C), indicating color variation close to pale yellow. Moreover, the pirarucu trimming surimi showed low color intensity (C*) and high whiteness, indicating adequate color quality.

**Figure 5:**
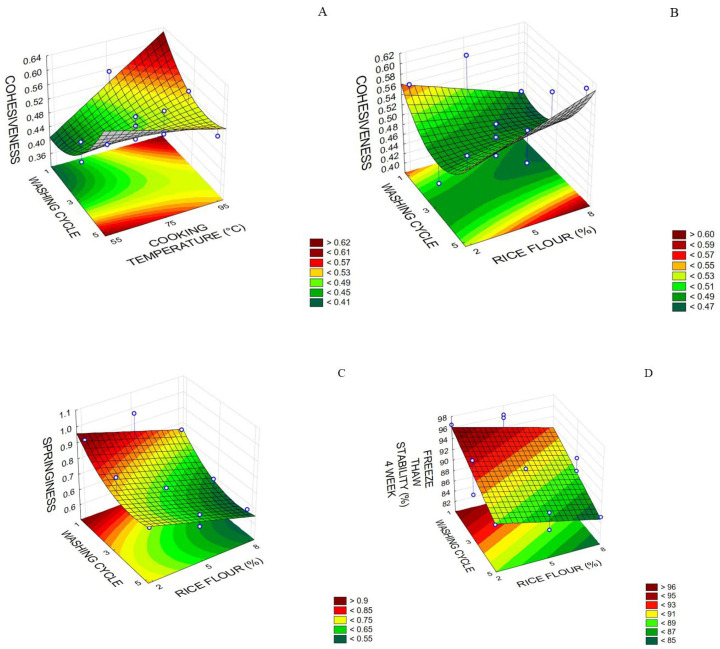
(**A**) Cohesiveness of the pirarucu (*Arapaima gigas*) trimming surimi concerning cooking temperature (°C) of rice flour (RF) and the number of washing cycles; (**B**) cohesiveness concerning RF concentration (%) and the number of washing cycles; (**C**) springiness concerning RF concentration (%) and the number of washing cycles; (**D**) freeze–thaw stability after 4 weeks concerning RF concentration (%) and the number of washing cycles.

Regarding the texture of pirarucu trimming surimi, the model could be adjusted only to cohesiveness and springiness (Table 5). As for cohesiveness, the number of washing cycles and RF cooking temperature displayed a positive linear effect and a positive quadratic effect for the number of washing cycles. Also, it was possible to verify interactions between the washing cycle and RF concentration and between the washing cycle and RF cooking temperature. For RF concentration set at 5%, a maximum cohesiveness value of 0.62 was obtained with one to two washing cycles and an RF cooking temperature between 85 and 95 °C, or four to five washing cycles and temperature between 55 and 65 °C (Figure 5A). For RF cooking temperature set at 70 °C, a maximum cohesiveness value of 0.60 was obtained with flour addition between 7% and 8% and four to five washing cycles (Figure 5B). The number of washing cycles and RF concentration showed a negative linear effect on springiness, RF cooking temperature showed a positive linear effect, and the number of washing cycles showed a quadratic effect, in addition to the interaction between the washing cycles and RF cooking temperature (Table 5). The maximum springiness value (0.9) can be obtained with 2% RF and one washing cycle at a fixed RF cooking temperature of 70 °C (Figure 5C).

Liu et al. [47] reported values from 0.67 to 0.69 for cohesiveness and values from 0.88 to 0.90 for springiness in silver carp surimi, with and without starch addition, values slightly higher than those verified for pirarucu trimming surimi. According to the authors, cohesiveness indicates sample recovery after the first compression, and springiness indicates how the original height was recovered after the first compression. Cohesiveness values close to 0.70 and a springiness of 0.90 indicate characteristics of viscoelastic materials, which is the case of surimi. Nevertheless, low viscoelasticity is desired for a restructured surimi product. Compared to the silver carp surimi mentioned above, pirarucu trimming surimi has low cohesiveness; thus, it requires less strength and is less elastic, being simple to shape for the preparation of the restructured product.

As for the freeze–thaw stability in 4 weeks, the number of washing cycles and RF content showed a negative linear effect (Table 5). With rice flour cooking temperature set at 70 °C, the maximum freeze–thaw stability value was 96% when the rice flour content varied between 2% and 3% and only one washing cycle was performed (Figure 5D). Jia et al. [12] worked with Alaska pollock surimi and added potato and wheat starch (5%), and they observed that drip loss was lower in surimi with small granule starch than native starch. Also, these authors found that after freezing and thawing, ice crystals melted and drip loss was released from surimi, yet this phenomenon was not observed in the preparation of pirarucu trimming surimi. One possible explanation is the higher moisture content (74.5%) and lower lipid content (0.4%) in Alaska pollock surimi compared with pirarucu surimi.

**Figure 6:**
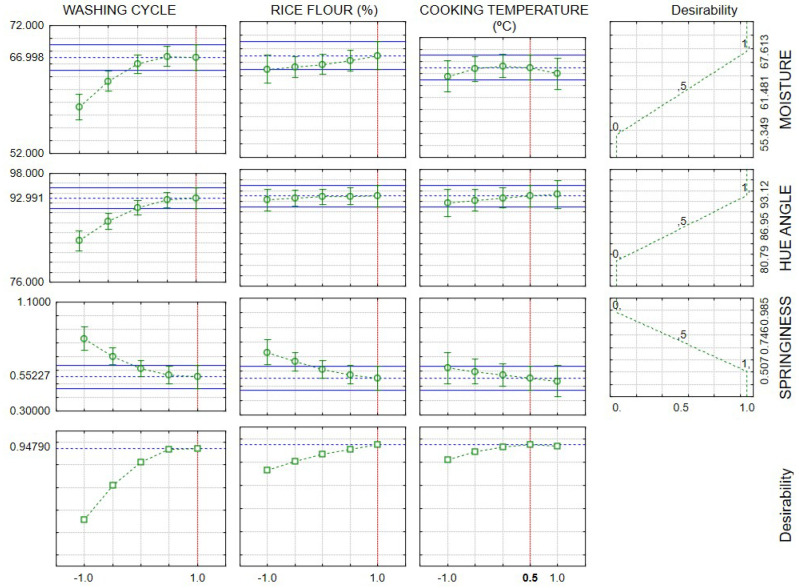
Desirability test of the properties of surimi manufactured with different washing cycles, rice flour levels (%), and gelatinization temperature (°C) considering higher moisture and hue angle, and lower springiness as more desirable.

The desirability test indicated that surimi had higher moisture and hue angle, and lower springiness values. Still, the desirability we obtained did not coincide with any test performed in the design. The graph revealed actual values for three washing cycles, 6% RF, and RF cooking temperature at 85 °C (Figure 6).

Considering that in the validation of the adequacy of mathematical models, a maximum error of 10% was set, it was found that only the models for yield, lipids, and springiness exceeded this limit (Table 6). Thus, the models for moisture, protein, whiteness, chroma, hue angle, cohesiveness, and freeze–thaw stability with 4 weeks of storage can be considered predictive.

Despite the prediction model for moisture, the experimental (66.27%) and predicted (62.17%) values indicate a low moisture level, making it impossible to obtain a high-quality surimi gel since a proper gelling requires a surimi paste with a moisture content of about 80% [48].

## 4. Conclusions

The number of washing cycles affected yield, moisture content, lipids and proteins, whiteness, chroma, hue angle, cohesiveness, springiness, and freeze stability after 4 weeks. Rice flour content acted upon moisture and lipid content, cohesiveness, springiness, and freeze–thaw stability after 4 weeks, whereas rice flour cooking temperature affected moisture, whiteness, chroma, cohesiveness, and springiness. The models were predictive for moisture, protein, whiteness, chroma, hue angle, cohesiveness, and freeze–thaw stability after 4 weeks of storage. It is possible to obtain an increase in moisture with the increase in the number of washing cycles and starch addition, allowing for the efficient removal of water-soluble proteins with three washing cycles, thus producing surimi with a tendency to appear pale yellow according to lipid content. The surimi with utmost desirability was processed with three washing cycles, 6% RF, and an RF cooking temperature of 85 °C. Despite the washings, pirarucu has a high yield for making surimi. The use of industrial by-products such as pirarucu trimmings and broken rice grains is feasible for the production of pirarucu trimming surimi with high yield and high physical, chemical, and technological quality, with the potential to be used as an ingredient in restructured products, in addition to contributing to improving agri-industry sustainability.

## Figures and Tables

**Table 1:** Experimental design used to obtain pirarucu (*Arapaima gigas*) trimming surimi, coded variables, and their actual values (X_1_: washing cycles; X_2_: rice flour—%; X_3_: cooking temperature—°C).

Experiments	Coded Variables			Actual Variables		
	X_1_	X_2_	X_3_	X_1_	X_2_	X_3_
1	−1	−1	0	1	2	70
2	−1	1	0	1	8	70
3	1	−1	0	5	2	70
4	1	1	0	5	8	70
5	0	0	0	3	5	70
6	−1	0	−1	1	5	55
7	−1	0	1	1	5	95
8	1	0	−1	5	5	55
9	1	0	1	5	5	95
10	0	0	0	3	5	70
11	0	−1	−1	3	2	55
12	0	−1	1	3	2	95
13	0	1	−1	3	8	55
14	0	1	1	3	8	95
15	0	0	0	3	5	70

**Table 2:** Thermal and pasting properties of rice flour.

Properties	Values
Gelatinization initial temperature ^1^	60.12 ± 0.53
Gelatinization peak temperature ^1^	63.88 ± 0.00
Gelatinization final temperature ^1^	69.71 ± 0.04
Gelatinization enthalpy (ΔH) ^2^	5.72 ± 0.06
Pasting temperature ^2^	65.20 ± 0.07
Peak viscosity ^3^	212.50 ± 0.82
Breakdown viscosity ^3^	169.67 ± 0.59
Tendency to retrogradation ^3^	148.13 ± 3.95
Final viscosity ^3^	317.79 ± 3.36

^1^ (°C); ^2^ (J/g); ^3^ (RVU).

**Table 3:** Results of yield, moisture, proteins, lipids, and color analysis, where x_1_ = wash cycle; x_2_ = rice flour (%); x_3_ = gelatinization temperature (°C).

Treatments	Actual Variables			Response Variables						
	x_1_	x_2_	x_3_	Yield (%)	Moisture (%)	Protein (%)	Lipids (%)	Hue (°)	Chroma (Dimensionless)	Whiteness (%)
1	1	2	70	90.26	56.00 ± 0.23	11.84 ± 0.59	27.31 ± 0.98	80.79 ± 0.54	12.86 ± 0.62	49.26 ± 2.23
2	1	8	70	89.88	59.73 ± 0.44	10.75 ± 0.42	29.38 ± 0.55	83.13 ± 0.85	13.18 ± 0.67	46.73 ± 2.25
3	5	2	70	87.57	64.87 ± 0.50	9.50 ± 0.20	34.65 ± 0.70	93.12 ± 0.67	9.80 ± 0.46	60.02 ± 1.72
4	5	8	70	80.29	67.61 ± 0.27	9.74 ± 0.24	31.24 ± 0.75	92.99 ± 0.84	9.00 ± 0.30	62.65 ±1.21
5	3	5	70	88.60	64.71 ± 0.18	10.17 ± 0.21	30.20 ± 0.78	88.98 ± 0.49	9.92 ± 0.54	58.95 ± 2.41
6	1	5	55	92.39	55.35 ± 0.54	11.55 ± 0.33	32.36 ± 0.55	81.95 ± 2.00	11.07 ± 0.41	55.86 ± 2.01
7	1	5	95	89.52	56.31 ± 0.10	11.88 ± 0.41	34.33 ± 1.69	83.83 ± 0.54	11.27 ± 0.41	55.68 ± 1.30
8	5	5	55	77.55	65.00 ± 0.59	10.42 ± 0.25	33.90 ± 0.87	91.99 ± 0.85	8.86 ± 0.40	64.45 ± 1.53
9	5	5	95	77.09	64.36 ± 0.94	9.09 ± 0.27	35.03 ± 0.04	92.46 ± 0.95	9.10 ± 0.29	63.00 ± 1.15
10	3	5	70	93.07	65.16 ± 0.48	7.15 ± 0.15	29.35 ± 6.87	90.37 ± 0.47	10.24 ± 0.44	58.50 ± 1.66
11	3	2	55	84.04	62.10 ± 0.35	8.90 ± 0.36	25.43 ± 0.67	89.39 ± 1.34	9.30 ± 0.44	63.24 ± 1.94
12	3	2	95	90.80	63.23 ± 0.85	9.94 ± 0.47	23.19 ± 1.19	88.68 ± 1.66	9.20 ± 0.48	62.52 ± 1.90
13	3	8	55	89.20	63.06 ± 0.35	8.82 ± 0.40	22.70 ± 0.39	89.39 ± 1.15	9.34 ± 0.40	61.90 ± 0.40
14	3	8	95	91.45	65.01 ± 0.52	8.80 ± 0.34	18.02 ± 0.88	91.48 ± 0.79	9.50 ± 0.35	59.14 ± 3.05
15	3	5	70	89.95	63.92 ± 0.84	9.16 ± 0.27	20.74 ± 0.14	90.89 ± 0.43	9.68 ± 0.41	60.59 ± 1.62

**Table 4:** Results of texture, freeze–thaw stability after 1 and 4 weeks, where x_1_ = wash cycle; x_2_ = rice flour (%); x_3_ = gelatinization temperature (°C).

Treatments	Actual Variables			Response Variables							
	x_1_	x_2_	x_3_	Hardness (N)	Elasticity (Ratio)	Cohesiveness (Ratio)	Gumminess (N)	Chewiness (N × mm)	Adhesiveness (Ratio)	1 Week (%)	4 Week (%)
1	1	2	70	4.89 ± 0.13	0.98 ± 0.01	0.56 ± 0.09	2.74 ± 0.39	2.68 ± 0.38	0.92 ± 0.13	96.67 ± 0.07	96.53 ± 0.15
2	1	8	70	4.52 ± 0.20	0.98 ± 0.01	0.49 ± 0.03	2.20 ± 0.15	2.15 ± 0.15	0.78 ± 0.06	90.46 ± 0.05	87.65 ± 2.03
3	5	2	70	5.00 ± 0.26	0.97 ± 0.03	0.55 ± 0.04	2.73 ± 0.15	2.64 ± 0.11	0.72 ± 0.06	89.24 ± 0.81	88.81 ± 2.00
4	5	8	70	4.27 ± 0.11	0.96 ± 0.02	0.61 ± 0.04	2.60 ± 0.12	2.50 ± 0.14	0.59 ± 0.07	88.63 ± 0.90	85.16 ± 0.48
5	3	5	70	5.16 ± 0.27	0.98 ± 0.02	0.45 ± 0.02	2.32 ± 0.23	2.27 ± 0,17	0.67 ± 0.07	81.79 ± 0.45	88.97 ± 2.54
6	1	5	55	6.15 ± 0.23	0.99 ± 0.00	0.40 ± 0.05	2.45 ± 0.25	2.44 ± 0.25	0.64 ± 0.07	96.11 ± 0.14	96.31 ± 0.39
7	1	5	95	4.21 ± 0.08	0.97 ± 0.02	0.59 ± 0.15	2.48 ± 0.60	2.40 ± 0.55	0.99 ± 0.14	96.08 ± 0.14	95.71 ± 0.32
8	5	5	55	4.46 ± 0.15	0.97 ± 0.02	0.56 ± 0.04	2.51 ± 0.27	2.45 ± 0.27	0.68 ± 0.03	82.91 ± 1.48	85.33 ± 1.25
9	5	5	95	5.43 ± 0.20	0.97 ± 0.02	0.50 ± 0.03	2.72 ± 0.22	2.62 ± 0.21	0.60 ± 0.05	91.22 ± 2.01	88.54 ± 1.26
10	3	5	70	5.19 ± 0.28	0.98 ± 0.03	0.49 ± 0.06	2.52 ± 0.28	2.47 ± 0.25	0.64 ± 0.06	90.34 ± 1.10	91.19 ± 0.69
11	3	2	55	3.93 ± 0.11	0.95 ± 0.00	0.43 ± 0.02	1.68 ± 0.07	1.60 ± 0.06	0.74 ± 0.06	86.36 ± 1.03	88.68 ± 0.38
12	3	2	95	6.31 ± 0.19	0.97 ± 0.02	0.48 ± 0.01	3.03 ± 0.14	2.93 ± 0.16	0.85 ± 0.14	90.49 ± 2.57	94.96 ± 0.03
13	3	8	55	3.96 ± 0.15	0.98 ± 0.02	0.48 ± 0.09	1.92 ± 0.40	1.88 ± 0.38	0.51 ± 0.07	87.83 ± 0.96	90.95 ± 1.13
14	3	8	95	4.55 ± 0.22	0.96 ± 0.01	0.55 ± 0.07	2.48 ± 0.29	2.38 ± 0.28	0.62 ± 0.04	90.16 ± 0.16	88.52 ± 0.98
15	3	5	70	6.05 ± 0.32	0.95 ± 0.01	0.51 ± 0.08	3.10 ± 0.45	2.95 ± 0.43	0.63 ± 0.10	88.19 ± 1.36	89.12 ± 1.96

**Table 5:** Adjusted model, significance level (p), correlation coefficient (R^2^), and lack of (LA) yield (%) for moisture (%), lipids (%), hue angle hue (°), chroma (dimensionless), freeze stability, cohesiveness, and springiness (mm) of pirarucu (*Arapaima gigas*) trimming surimi submitted to different washing cycles (x_1_), rice flour (RF) concentration (%; x_2_), and cooking temperature of RF (°C; x_3_). The models use coded levels of the variables.

Responses	Adjusted Model	p	R^2^	LA
Moisture	y = 64.77 + 4.31x_1_ − 2.84 x_1_^2^ + 1.15x_2_ − 1.54x_3_^2^	0.0039	0.97	0.41
Lipids	y = 26.40 + 7.77 x_1_^2^ − 3.80x_2_^2^	0.038	0.71	0.96
Proteins	y = 11.213 − 0.456x_1_	0.000	0.86	0.99
Whiteness	y = 58.51 + 5.32x_1_ − 3.21x_1_^2^ + 3.82x_3_^2^	0.022	0.89	0.24
Chroma	y = 10.11 − 1.45x_1_ + 0.98 x_1_^2^ − 0.89x_3_^2^	0.0016	0.93	0.32
Hue angle	y = 80.97 + 2.56x_1_	0.000000	0.95	0.57
Cohesiveness	y = 0.483 + 0.022x_1_ + 0.049x_1_^2^ + 0.030x_3_ + 0.034x_1_x_2_ − 0.063x_1_x_3_	0.024	0.82	0.60
Springiness	y = 0.665 − 0.091x_1_ + 0.075x_1_^2^ − 0.092x_2_ + 0.062x_3_ − 0.106x_1_x_3_	0.00038	0.96	0.33
Freeze stability	y = 90.43 − 3.54x_1_ − 2.09x_2_	0.025	0.68	0.22
yield	y = 94.86 − 2.4717x_1_	0.003	0.51	0.36

**Table 6:** Actual values, predicted values (model), and percentage error of validation assay for yield, moisture, lipids, proteins, whiteness, chroma (C*), hue angle, cohesiveness, springiness, and freeze–thaw stability.

Parameters	Actual Value (Experimental)	Model Value (Predicted)	% Error
Yield (%)	74.73	89.59	20%
Moisture (%)	66.27	62.17	6%
Lipids (%)	15.93	20.93	31%
Proteins (%)	8.38	8.99	7%
Whiteness (%)	56.15	61.54	10%
Chroma (dimensionless)	10.45	9.41	10%
Hue angle (°)	93.08	89.88	3%
Cohesiveness (ratio)	0.46	0.46	1%
Springiness (ratio)	0.47	0.72	54%
Freeze–thaw stability after 4 weeks (%)	91.60	92.94	1%

## Data Availability

Data are available on request from the authors.
